# Egyptian recommendations for the management of systemic lupus erythematosus: a consensus, evidence-based, clinical practice guidelines for treat-to-target management

**DOI:** 10.1186/s43166-023-00187-9

**Published:** 2023-04-06

**Authors:** Yasser El Miedany, Khaled Elhadidi, Geilan Abdelmoneim Mahmoud, Mohammed Hassan Abu-Zaid, Atef Abdelazim Mahmoud, Maha El Gaafary, Nadia Kamel, Nihal Ahmed Fathi, Ahmed Abdel Nasser, Waleed Hassan, Mervat Eissa, Eman Sarhan, Essam Aboalfadl, Ahmed Ezzat Mansour, Mohamed Mortada, Nermeen Ahmed Fouad, Ismail Elaraby, Rehab Elnemr, Basma M. Medhat, Sally S. Mohamed, Rehab Ali Ibrahim, Samar abd Alhamed Tabra, Sally Saber, Genny Franklin, Abir Mokbel

**Affiliations:** 1 Canterbury Christ Church University, Canterbury, England; 2 Rheumatology, Cairo University, Cairo, Egypt; 3 Rheumatology, Tanta University, Tanta, Egypt; 4 Internal Medicine & Rheumatology, Cairo University, Cairo, Egypt; 5 Community and Public Health, Ain Shams University, Cairo, Egypt; 6 Physical Medicine, Rheumatology & Rehabilitation, Faculty of Medicine, Ain Shams University, Cairo, Egypt; 7 Rheumatology and Rehabilitation, Assiut University, Assiut, Egypt; 8 Rheumatology & Rehabilitation, Minia University, Minia, Egypt; 9 Rheumatology and Rehabilitation, Benha University, Benha, Egypt; 10 Nephrology, Ain Shams University, Cairo, Egypt; 11 Rheumatology, Sohag University, Sohag, Egypt; 12 Internal Medicine and Nephrology, Benha University, Benha, Egypt; 13 Rheumatology and Rehabilitation, Zagazig University, Zagazig, Egypt; 14 Rheumatology and Rehabilitation, Fayoum University, Fayoum, Egypt; 15 Ministry of Health, Cairo, Red Sea Government Egypt; 16 Physical Medicine, Rheumatology and Rehabilitation, Alexandria University, Alexandria, Egypt; 17 Rheumatology and Rehabilitation Department, Faculty of Medicine Al-Kasr Alainy, Cairo University, Cairo, Egypt; 18 Rheumatology and Rehabilitation, Cairo University, Cairo, Egypt; 19 Rheumatology and Rehabilitation, Ain Shams University, Cairo, Egypt; 20 Rheumatology and Rehabilitation, Tanta University, Tanta, Egypt; 21 Dartford & Gravesham Library, Kent, UK

**Keywords:** Systemic lupus eryhtematosus, Discoid lupus erythematosus, SLE, Treat-to-target, PICO, Outcomes, Lupus, Diagnosis, Assessment, Monitoring, Management, Immunosuppressants, Treatment, Efficacy, Non-biologics, Biologics, Egyptian guidelines

## Abstract

**Background:**

Systemic lupus erythematosus (SLE) is a chronic systemic autoimmune disease characterized by having varying clinical presentation, severity, unpredictable course as well as outcomes. Recent disease-modifying conventional and biologic agents have enhanced rates of attaining both short- and long-term management goals, including minimization of glucocorticoid dose and use. This study was carried out to develop an up-to-date evidence-based, consensus on clinical practice guidelines for treat-to-target management of systemic lupus erythematosus in adults.

**Results:**

The response rate to the online questionnaires, sent to the expert panel who participated in the three rounds, was 95.5%. At the end of round 3, a total of 14 recommendation sections were proposed for the T2T management of patients with SLE. Agreement with the recommendations (rank 7–9) ranged from 90.9–100%. Consensus was reached (i.e., ≥ 80% of respondents strongly agreed or agreed) on the proposed statements.

**Conclusion:**

These recommendations provide a consensus on the treat-to-target management of patients with SLE. They provide strategies to reach optimal outcomes in common clinical scenarios, based on a combination of evidence and expert opinion.

## Background

Systemic lupus erythematosus (SLE) is a multi-system autoimmune disease that can affect different organ systems with consequent organ damage and poor health related quality of life [1]. The estimated prevalence of SLE in Egypt in adults has been estimated to be 6.1/100,000 population (1.2/100,000 males and 11.3/100,000 females) according to a multi-center, nation-wide study done on Egyptian patients in 2021. However, taking into consideration unregistered patients presenting to private clinics and early undiagnosed cases, the estimate would be much higher reaching 2–3 times more [2]. Disease activity in SLE has different trajectories whether relapsing remitting, long quiescent, chronically active disease. SLE management should aim at disease control, and prevention of disease flare [3]. Controlling disease activity will prevent further damage and improve the disease outcomes [4].

Treat-to-target approach is a therapeutic strategy in which treatment modification is done to achieve a certain predefined goal [5]. Setting a pre-specified target to reach for disease activity control that could be quantitatively assessed is a concept adopted for better control of the disease flare. These treatment target points were found to be associated with less damage and better health related quality of life, and it was also associated with better long-term outcomes [6]. This concept which is borrowed from other rheumatological and non-rheumatological diseases will help health care providers to optimize care of this group of patients with early identification of patients in need for a therapeutic change [7]. Analyses from several cohorts demonstrate that staying in remission or lupus low disease activity state (LLDAS) is associated with a favorable outcome [8]. There are several unmet needs in SLE management in developing countries including Egypt. Factors contributing to this include high percentage of illiterate population, low socioeconomic class, low availability of health care resources along with other factors [9].

The objective of this work was to set up an actionable clinical gold standard management for SLE in Egypt through developing a consensus on the evidence-based treat-to-target guidance for management of SLE patients.

## Methods

### Development stages

The study was done on two stages: (1) a qualitative synthesis of scientific evidence through literature review to develop evidence-based treat-to-target guidance for management of patients with SLE. (2) The results were redrafted as recommendation and the level of agreement between clinical experts on these recommendations were evaluated. The study was managed by a core team. It was formed of three experts with recognized experience in SLE management. The core team supervised and coordinated the teamwork. They assisted the development of the clinical questions which guided the literature search. They managed the literature review process. The core team also nominated the expert panel who shared in the voting process.

## Literature review and building the statements

The literature search was done by a knowledgeable information specialist based on the specific key questions (Table 1) which were specified by the core team and the literature review team. They were structured according to the PICO format (population, intervention, comparison, and the outcome). The following databases were searched from inception to September 18th 2021: (1) MEDLINE, (2) Embase, Keywords, Medical Subject Headings (MeSH), and Emtree subject headings related to SLE, SLE management were used to build the search.

**Table 1:** Key clinical questions

1. Who are the targeted population?
2. What are the definitions of mild, moderate and severe lupus? (assessment of disease activity and severity)
3. What are the treatment targets?
4. How should SLE patients be monitored in the non-acute setting?
5. What is the frequency of monitoring lupus/follow-up visits?
6. What are the non-pharmacological and preventive interventions?
7. What is the evidence for the management of mild SLE?
8. What is the evidence for the management of moderate SLE?
9. What is the evidence for the management of severe SLE?
10. What are the recommendations for management of acute emergencies in patients with SLE (severe neurologic involvement, systemic vasculitis, profound thrombocytopenia with a thrombotic thrombocytopenia (TTP)–like syndrome, rapidly progressive glomerulonephritis, Diffuse alveolar hemorrhage)
11. What are the recommendations for Specific organ system involvement in SLE?
12. What are the recommendations for treating the associated comorbid conditions?
13. What are the prognostic markers?
14. What are the recommendations for the management of refractory patients?

Search results were managed using an excel software. Two reviewers independently screened the title and abstracts of the retrieved articles to identify relevant studies. They then independently screened the full texts of the relevant articles. In addition, reference lists of the relevant articles were examined to identify additional studies. Conflicts were resolved through discussion.

### Inclusion criteria

Articles included were systematic reviews, randomized controlled trials (RCTs), uncontrolled trials, cohort, case-control, and cross-sectional studies. The search was limited to studies with adult SLE subjects ≥ 18 years old, regardless of sex, health care setting, or treatment.

### Exclusion criteria

Articles discussing the diagnosis and investigations for SLE patients were excluded. Articles for economic evaluation, editorials, commentaries, conference abstracts were excluded. Articles not written in English were excluded.

Search results were screened for title and abstract first for preliminary inclusion. The full texts of the selected articles were then screened. Based on the results of the literature review, comprehensive statements answering each proposed clinical question were prepared. The level of evidence was determined for each section using the Oxford Centre for Evidence-based Medicine (CEBM) system [10] (Table 2).

**Table 2:** Levels of evidence

Level of evidence	
1	Systematic review of all relevant randomized clinical trials or *n*-of-1 trials
2	Randomized trial or observational study with dramatic effect
3	Non-randomized controlled cohort/follow-up study (observational)
4	Case series, case–control study, or historically controlled study
5	Mechanism-based reasoning (expert opinion, based on physiology, animal, or laboratory studies)
Grades of recommendation	
A	Consistent level 1 studies
B	Consistent level 2 or 3 studies, or extrapolations from level 1 studies
C	Level 4 studies, or extrapolations from level 2 or 3 studies
D	Level 5 evidence or troubling, inconsistent or inconclusive studies of any level

## Evaluating the consensus

For evaluating the consensus on the proposed statements, the core leadership team nominated the expert panel participants. The criteria for their selection included practice in the Egyptian health system, have professional knowledge and experience (at least 8 years of experience) in management of inflammatory arthritis and in particular SLE as well as active participation in scientific research on SLE. The proposed recommendation statements were sent to the expert panel for voting. Three Delphi rounds was carried out to establish a consensus. We used the Delphi process due to the anonymity of the participants and the controlled feedback [11–13]. The structured Delphi approach ensured that the opinions of participants were equally considered, and it was particularly useful for geographically diverse centres as in Egypt. The first round of the electronic questionnaire included 14 questions involved in the treat-to-target (T2T) strategy of SLE. The second and the third rounds included 14 sections. The expert panel was invited to participate and was pre-informed of the time of opening and closure of each round of votes. Anonymous votes were gathered and processed. Comments on re-phrasing, potential ambiguity, unidentified overlaps were gathered regarding each statement at the same time in the voting process.

Each statement was rated between 1 and 9 with 1 being ‘complete disagreement’ and 9 being ‘complete agreement’. Generally, 1–3, 4–6, and 7–9 represent disagreement, uncertainty, and agreement, respectively. There was no requirement to vote on all statements, and the members are encouraged to abstain if they feel that a statement falls outside their area of expertise. Therefore, an ‘uncertainty’ vote represents ‘inconvenience about the accuracy of the recommendation’. All the statements were allowed for the entry of comments which will be reviewed by the scientific committee after each round of voting. In all the votes’ rounds, the members are further urged to leave comments wherever they vote a disagreement. This enabled the core team to identify an instance of misinterpretation of statement and invalidate the vote on that statement.

### Definition of consensus

Definition of consensus was established before data analyses. It is determined that consensus, consequently, to become a recommendation in this guideline, would be achieved if at least 75% of participants reached agreement (score 7–9) or disagreement (score 1–3) [11]. A statement is retired if it got a mean vote below 3 or a ‘low’ level of agreement. Statements whose rate came in the uncertainty score, (4–6), were revised in view of the comments. The levels of agreement on each statement of recommendation were defined as ‘high’ if after the third round of votes, all votes on a statement fell into the agreement bracket (7–9) [11].

### Voting process

The Delphi process was conducted through online questionnaires. The first round of the electronic questionnaire included 14 questions involved in the T2T strategy of SLE.

All members were pre-informed of the time of the opening and the closure of each round of votes. Access links were sent out, and anonymous votes were gathered and processed. Comments on re-phrasing, potential ambiguity were collected regarding each statement at the same time in the voting process.

### Chronogram of Delphi rounds

The first round took place between 23rd April and 28th April 2022 (6 days). The aspects about which respondents did not reach consensus in this first round were revised in view of the comments and included in the second round; the response rate on the first round was 95.5%. The second round took place on 29th of May 2022 and lasted for 6 days (till 3rd of June 2022), the response rate on the 2nd round was 100%. Regarding 3rd round; the response rate was 100%, and it took place between 3rd and 8th August 2022.

### Ethical aspects

This study was performed in accordance with the Helsinki Declaration. The Clinical, Evidence-based, Guidelines” (CEG) initiative protocol was approved by the ethical committee board, Tanta University: ethical approval code: 34842/8/21.

## Results

### Literature research and evidence selection

**Fig. 1:**
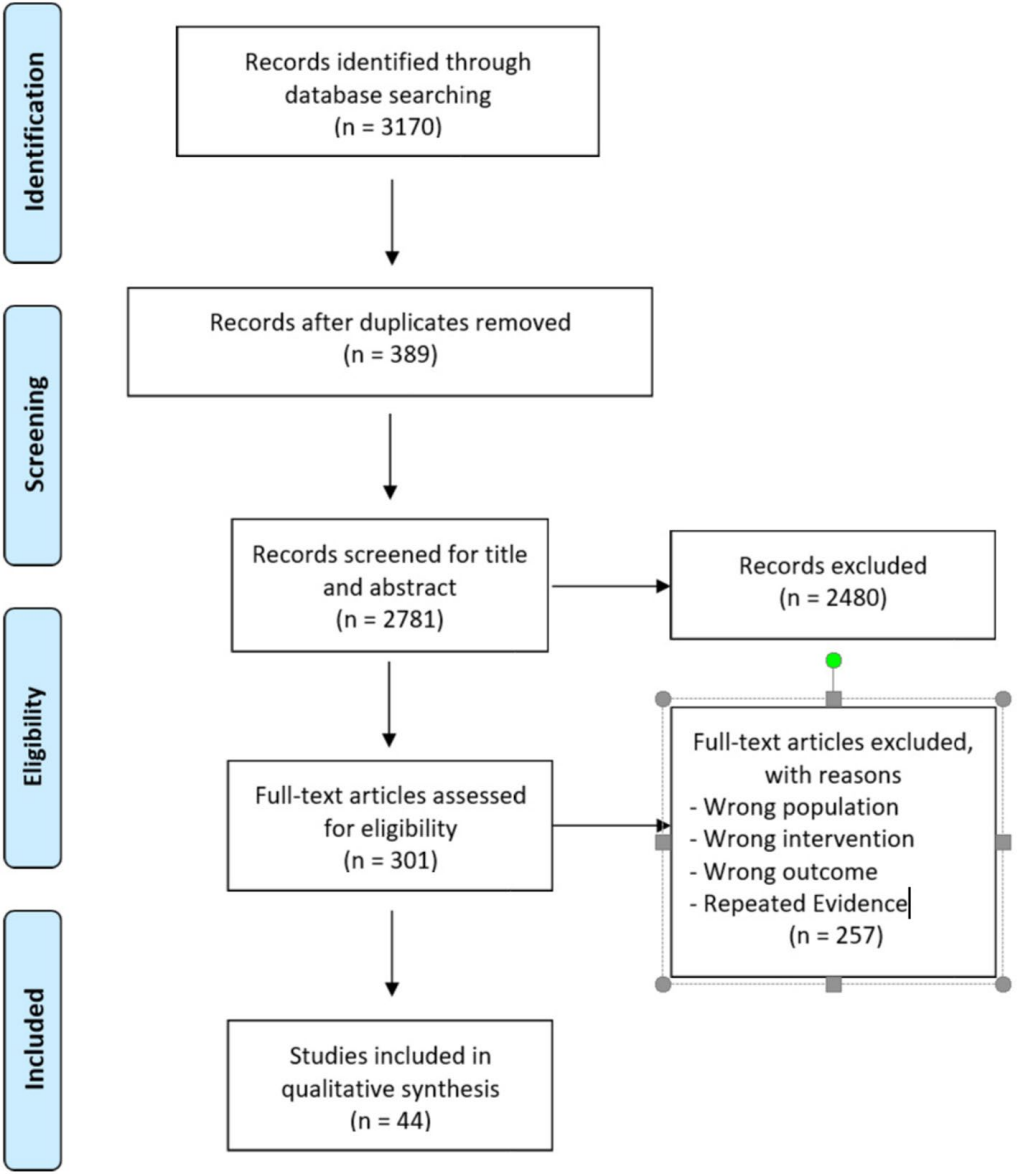
Flow diagram of the studies’ selection process

The search yielded 3170 records, of which 2781 were screened after removing the duplicates (389). After screening, we retrieved the full text of 301 potential studies. Forty-four articles were included in the literature review. The study selection process is represented as a PRISMA diagram in Fig. 1.

The results were collected, condensed, and formulated as recommendations of management of patients with SLE. They then were discussed, modified, and voted upon.

### Expert panel characteristics

The Delphi form was sent to expert panel (*n* = 22), of whom 21 (95.5%) completed the first round, while 22 (100%) completed in the 2nd and 3rd rounds. The participants were from governorates and health centres throughout Egypt: Ain Shams University (*n* = 4, 18.18%), Cairo University (*n* = 7, 31.82%), Tanta University (*n* = 2, 9.09%), Benha University (*n* = 2, 9.09%), Alexandria University (*n* = 1, 4.55%), Fayoum University (*n* = 1, 4.55%), Sohag University (*n* = 1, 4.55%), Zagazig University (*n* = 1, 4.55%), Assiut University (*n* = 1, 4.55%), and Minia University (*n* = 1, 4.55%), in addition to (*n* = 1, 4.55%) international expert from UK. All the experts’ panel were rheumatologists.

#### Delphi round 1

This round was dedicated to the key clinical questions, which included 14 items (Table 1) including: all clinical questions which answered in the subsequent rounds. All domains and questions were agreed upon (with 80% of respondents strongly agreeing or agreeing), and no questions were retired.

#### Delphi round 2

Based on the literature research, a list of 14 sectioned recommendations were generated using the input from round 1. The response rate for round 2 was 100% from the experts’ panel (22/22). Wording modifications were suggested for 12 statements. The statements were modified and amended. For all statements, the consensus was reached (as ≥ 80% of respondents strongly agreed or agreed).

#### Delphi round 3

For the statements which had low level of agreement or comments.

### Statements and grade of recommendations (GOR) for the management of SLE

**Fig. 2:**
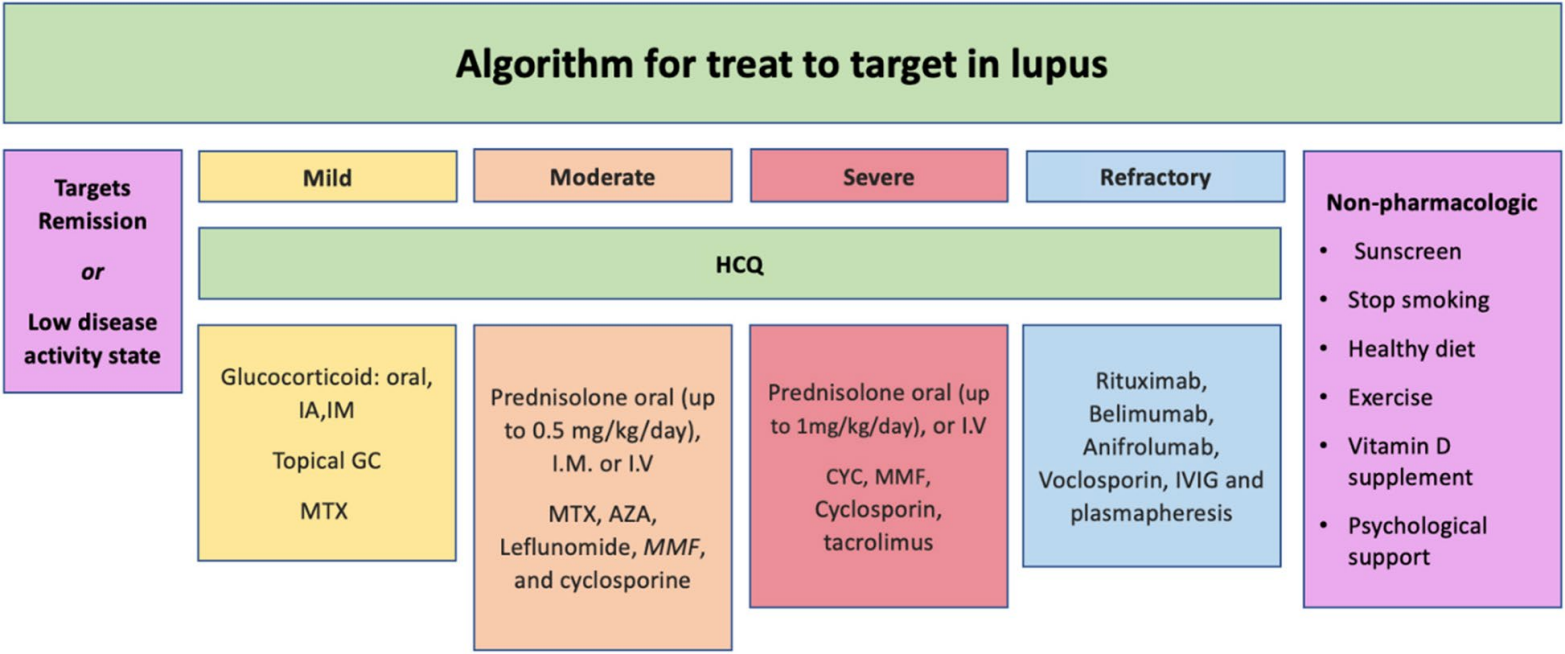
Algorithm for the outlines of treat to target in lupus patients

**Fig. 3:**
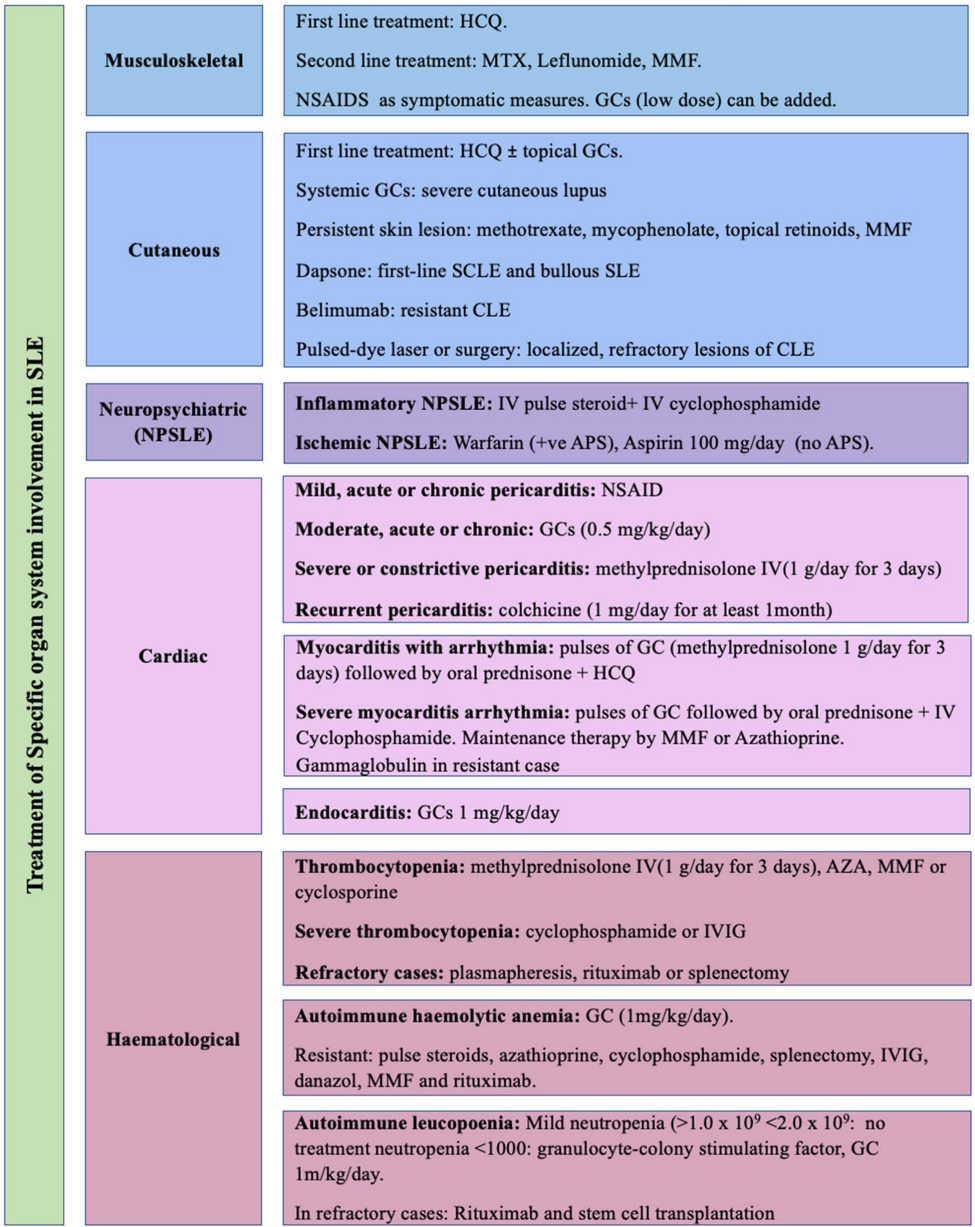
Treatment of specific organ system involved in SLE

**Fig. 4:**
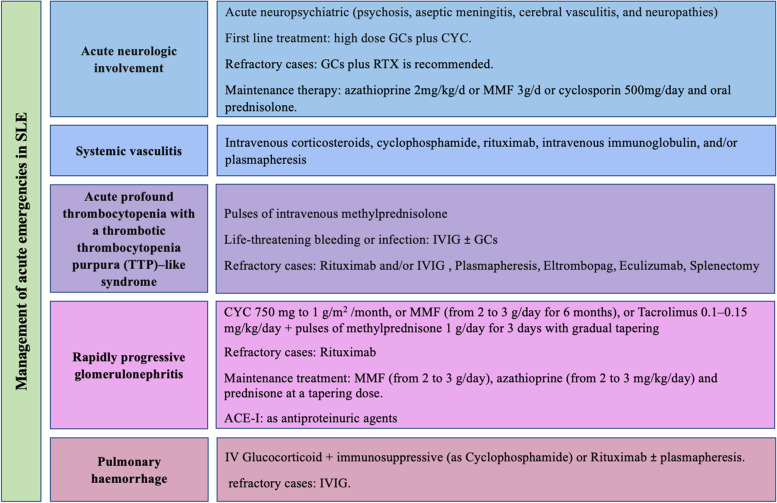
Management of acute emergencies in SLE

The recommendations formulated to answer the key clinical questions are listed below. The mean level of agreement between the members of the expert panel, percentage of agreement, the level of evidence (LOE), as well as grades of recommendations are mentioned under each section. An algorithm of these recommendations is demonstrated in Fig. 2. Figure 3 shows summary of the recommended treatment of specific organ system involved in SLE, whereas Fig. 4 shows suggested management approach of acute emergencies in SLE.

#### Overarching principles: [12, 13]

Mean rate ± SD: 8.9 ± 0.29, % of agreement: 100%, LOE: High

- The target audience of these recommendations includes rheumatologists and other clinicians who may have role in management of adult SLE patients such as nephrologists, immunologists, dermatologists, hematologist, and emergency medicine physicians.
- Management of SLE necessitates interdisciplinary care and shared-decisions making between SLE patients and their physicians. (LOE 2, GOR B)
- Patients with SLE should receive education, counseling, and support, particularly in terms of managing the complexity and unpredictability of the disease. (LOE 4, GOR C)
- Tight disease control with treat-to-target strategy should be adopted in SLE management. (LOE 3, GOR C)
- Treatment should not be given or modified for serological activity alone. (LOE 3, GOR C)

* Disease activity status:

Q. What are the definitions of mild, moderate and severe, and refractory lupus? [14, 15]

Mean rate ± SD: 8.9 ± 0.29, % of agreement: 100%, LOE: H

- Mild disease activity reflects clinically stable disease with no life-threatening organ involvement (fatigue, malar rash, diffuse alopecia, mouth ulcers, arthralgia, myalgia, platelets 50–149 × 109/l) and is not likely to cause significant scarring or damage, and/or SLEDAI-2 K score of < 6. (LOE 1, GOR A)
  -Moderate disease includes more serious manifestations, which if left untreated would cause significant chronic scarring (fever, lupus-related rash up to 2/9 body surface area, cutaneous vasculitis, alopecia with scalp inflammation, arthritis, pleurisy, pericarditis, hepatitis, platelets 25–49 × 109/l) and/or SLEDAI-2 K score in the range of 6–12. (LOE 1, GOR A).
  -Severe disease is defined as organ or life threatening manifestations which reflect the most serious form of systemic disease that requires potent immunosuppression (nephritis, central nervous system manifestations myelopathy, pneumonitis, mesenteric vasculitis, pulmonary hemorrhage, myocarditis, lupus pancreatitis, severe scleritis, rash involving > 2/9 body surface area, myositis, severe pleurisy, and/or pericarditis with effusion, ascites, enteritis, optic neuritis, platelets < 20 × 109, acute hemophagocytic syndrome) and/or a SLEDAI-2 K score of > 12 (LOE 1, GOR A).
- Refractory lupus could be defined as a persistent state of disease activity (SLEDAI-2 K > 6) with the presence of any of the following: (i) failure to respond to ≥ 2 documented immunosuppressives despite adequate treatment adherence, (ii) or the dependence on moderate or high glucocorticoids (40 mg or more) doses to control recurrent flares or persistent disease activity.
- NB: SLEDAI-2 K was used to stratify disease severity; however, other disease activity indices can be used. (LOE 1, GOR A).

* Treatment targets:

Q. What are the treatment targets? [16]

Mean rate ± SD: 8.77 ± 0.69, % of agreement: 95.5%, LOE: H

- Remission and low disease activity state (LDAS) would be the main pre-specified targets for better disease control. (LOE 4, GOR C)

* Patient monitoring:

Q. How should SLE patients be monitored in non-acute setting? [17]

Mean rate ± SD: 8.5 ± 0.96, % of agreement: 95.5%, LOE: H

- Patients with SLE should be monitored on a regular basis for disease activity, organ damage, health related quality of life, drug toxicity, and co-morbidities. (LOE 2, GOR A)
- The patients should be monitored for disease activity, using lupus-specific disease activity indices such as SLE DAI, SLEDAI-2 K, and SLEDAS. (LOE 2, GOR A)
- Organ damage can be monitored using the SLICC/ACR Damage Index. (SDI) (LOE 2, GOR A)
- Assessing patients ‘reported outcomes measures (PROM) in SLE patients can measure relevant aspects of health-related quality of life, symptoms, and functioning from patents’ own perspectives. PROM assessment can be done preferably by using disease specific tools such as Lupus QOL questionnaire. (LOE 2, GOR A)
- Laboratory monitoring of SLE includes full blood count, liver function tests, renal function tests, urinalysis, complement C3/C4, anti-dsDNA, C-reactive protein, serum albumin. Twenty-four-hour urinary proteins, urinary protein:creatinine ratio, eGFR are recommended when required (LOE 2, GOR A).
- Monitoring of drug induced side effects and comorbidities (such as infection, premature cardiovascular and peripheral vascular disease, osteoporosis, avascular necrosis, and some malignancies) should be considered as a crucial part of the management for HCQ toxicity monitoring, ophthalmological screening (by visual fields examination and/or spectral domain-optical coherence tomography) should be performed at baseline, after 5 years, and yearly thereafter or more frequently in the presence of risk factors for retinal toxicity, e.g., previous retinal disease, older age, hepatic, or renal impairment (LOE 2, GOR B).

Q. What is the frequency of monitoring lupus/follow-up visit? [18]

Mean rate ± SD: 8.5 ± 1.71, % of agreement: 95.5%, LOE: H

- Patient with active disease should be reviewed at least every 1–3 months (LOE 2, GOR B).
- For most patients with mild features, including those who are clinically quiet but serologically active, 3 monthly visits are adequate. (LOE 2, GOR B)
- Patients with stable low disease activity or in remission without previous renal involvement or organ damage can be reviewed less frequently, for example, 3–6 monthly (LOE 5, GOR D)
- Additional evaluation is recommended prior to pregnancy, surgery, organ transplantation, use of estrogen-containing medications, or occurrence of a new neurologic or vascular event. (LOE 3, GOR A)
- Monitoring of specific conditions as the presence of APLs (even if previously negative) is recommended. APLs should be re-evaluated prior to pregnancy or surgery, or in the presence or vascular/thrombotic event. (LOE 4, GOR D)

### Management

Q. What are the non-pharmacologic and preventive interventions? [19]

Mean rate ± SD: 8.86 ± 0.35, % of agreement: 100%, LOE: H

- Non-pharmacologic and preventive interventions include avoiding exposure to sunlight, smoking cessation, healthy diet (low calorie or glycemic index diet) and routine exercise. (LOE 2, GOR B)
- Sunscreen must be used in all cases to prevent flare (cutaneous and systemic) (LOE 2, GOR B)
- Vitamin D supplement is recommended and dose should be adjusted according to the patients’ serum level. (LOE 2, GOR C)
- Seeking for psychological support if needed, self-management for fatigue, and management of sleep disturbance are recommended. (LOE 2, GOR C)

Q. What is the evidence for the management of mild SLE? [20, 21]

Mean rate ± SD: 8.31 ± 1.7, % of agreement: 95.5%, LOE: H

- HCQ are recommended for management of mild SLE (LOE 1, GOR A)
- Short courses of oral prednisolone (up to 20 mg/day) are used for short periods of time (up to 14 days) and reduced gradually until reaching the lowest effective maintenance dose (recommended to be ≤ 7.5 mg/day) to induce remission in some cases of mild lupus (LOE 2, GOR B).
- Topical GC preparations should be used for cutaneous manifestations, and (IA) or (I.M.) injections of CSs can be used for arthritis. (LOE 3, GOR C)
- MTX can be used in low-dose weekly MTX (> 25 mg/week) if no hematological or renal contraindications to control inflammatory arthritis and lupus skin rashes if HCQ and low-dose CSs failed, but it can be used with HCQ to avoid CSs or to promote CS dose reduction. (LOE 1, GOR A)
- Short courses of NSAIDs for symptomatic control (inflammatory arthralgia, myalgia, chest pain, and fever) can be used. (LOE 4, GOR C)
- Management should aim at reducing and stopping all the drugs except HCQ eventually when in stable remission. (LOE 3, GOR C)

Q. What is the evidence for the management of moderate SLE? [22–26]

Mean rate ± SD: 8.31 ± 1.7, % of agreement: 95.5%, LOE: H

- Higher doses of prednisolone (up to 0.5 mg/kg/day) (LOE 2, GOR C), or the use of I.M. (LOE 4, GOR D) or I.V. doses of methylprednisolone (LOE 3, GOR C) are recommended. For chronic maintenance treatment, GC should be minimized to less than 7.5 mg/day (prednisone equivalent), and when possible, they should be withdrawn (LOE 1, GOR B).
- HCQ should be used in all cases prevent flare and as steroid sparing drug unless contraindicated (LOE 1, GOR A).
- Immunosuppressive agents such as MTX, (LOE 2, GOR B) AZA (LOE 3, GOR C), Leflunomide, MMF (LOE 2, GOR B), and cyclosporine (LOE 2, GOR B) are often required to control active disease and are steroid-sparing agents (LOE 3, GOR C). They can also reduce the risk of long-term damage accrual (LOE 3, GOR C). Prompt initiation of immunomodulatory agents can expedite the tapering/discontinuation of GC (LOE 3, GOR B).

Q. What is the evidence for the management of severe SLE [27–29]?

Mean rate ± SD: 8.72 ± 0.63, % of agreement: 100%, LOE: H

- Patients who present with severe SLE, including renal and neuropsychiatric manifestations, need thorough investigation to exclude other aetiologies, such as infection (LOE 5, GOR D).

Treatment is dependent on the underlying aetiology (inflammatory and/or thrombotic), and patients should be treated accordingly with immunosuppression and/or anticoagulation, respectively. (LOE 5, GOR D)

- Flares of SLE can be treated according to the severity of organ(s) involvement by adjusting ongoing therapies (glucocorticoids, immunomodulating agents) to higher doses, switching or adding new therapies. (LOE 3, GOR C)
- Immunosuppressive regimens for severe active SLE involve
  - IV methylprednisolone (LOE 3, GOR C) or high-dose oral prednisolone (up to 1 mg/kg/day) (LOE 5, GOR D) to induce remission, either on their own or more often as part of a treatment protocol with another immunosuppressive drug (LOE 5, GOR D).
  - CYC are used for most cases of severe LN and for severe non-renal disease (NPS manifestations, and severe immune thrombocytopenia
  - MMF can be used in severe renal (LOE 1, GOR A)) and non-renal lupus activity (musculoskeletal, cutaneous, hematological, and serological but not neuropsychiatric lupus) (LOE 3, GOR D).
  - Cyclosporin 3–5 mg/kg/day (100–400 mg/day) in 2 doses at the same time every day with meal or between meals (LOE 2, GOR B) and tacrolimus 0.07 mg/kg/day for lupus nephritis in 2 doses at the same time every and 1 mg twice daily SLE with immune thrombocytopenia.

Q. What are the recommendations for the management of refractory patients? [30–32]

Mean rate ± SD: 8.86 ± 0.35, % of agreement: 100%, LOE: H

- Rituximab (anti-CD20 mAb) is used for refractory non-renal and renal manifestations (LOE 3, GOR C).
- Belimumab (anti-Blys mAb): was approved for refractory non-renal involvement and as an add-on to SLE renal therapies (LOE 3, GOR C).
- Anifrolumab, a type I interferon (type I IFN) receptor antagonist, has been approved for treatment of moderate to severe SLE. (LOE 3, GOR C).
- Targeting cellular signaling such as Voclosporin (Calcineurin inhibitor) is the first approved oral therapy for treatment of LN (LOE 2, GOR C)
- IVIG (LOE 3, GOR C) and plasmapheresis (LOE 4, GOR C) may be considered in patients with refractory cytopenia, thrombotic thrombocytopenic purpura (TTP), rapidly deteriorating acute confusional state and the catastrophic variant of APS. (LOE 3, GOR B).

Q. What are the recommendations for specific organ system involvement in SLE? [33–38] (Fig. 3)

Mean rate ± SD: 8.68 ± 0.56, % of agreement: 100%, LOE: H

### Musculoskeletal manifestation

NSAIDS are recommended as symptomatic measures. First line treatment is HCQ. If needed, GCs (low dose) can be added. In case of activity, MTX, Leflunomide, MMF are second line treatment. (LOE 3, GOR C).

### Cutaneous lupus erythematosus (CLE)

- First-line treatment of skin disease in SLE includes topical GCs agents in people with localized CLE (including the face) for up to 4 weeks in addition to systemic therapy in more wide spreads disease. (LOE 3, GOR C)
- HCQ can be used as first line treatment option in CLE with or without topical steroids. Higher doses of HCQ with maximum dose of 5 mg/kg in severe cutaneous lupus or risk of scarring, e.g., discoid lupus can be used
- Systemic GCs in severe cutaneous lupus or risk of scarring, e.g., discoid lupus is recommended. If persistent skin disease (inadequate response to HCQ and topical steroids), methotrexate, mycophenolate, and topical retenoids can be used. (LOE 3, GOR C)
- Consider MMF in combination with HCQ in people with CLE with partial response to topical therapy and HCQ. (LOE 3, GOR C)
- Dapsone can be considered in a first-line systemic treatment option in people with SCLE and bullous SLE and 2nd line treatment CLE. Pulsed-dye laser or surgery can be used for localized, refractory lesions of CLE. (LOE 3, GOR C)
- Belimumab is considered in CLE treatment when conventional systemic therapies have failed. (LOE 4, GOR C)
- In cutaneous lupus there is insufficient evidence for the use of rituximab in CLE. (LOE 3, GOR C)

### Neuropsychiatric SLE

- Diffuse neuropsychiatric syndromes include aseptic meningitis, demyelinating syndrome, headache, acute confusional state, anxiety disorder, cognitive dysfunction, mood disorder, and psychosis. Focal NP syndromes include cerebrovascular disease, Guillain–Barre syndrome, movement disorder, myelopathy, seizure disorders, autonomic neuropathy, mononeuropathy, myasthenia gravis, cranial neuropathy, plexopathy, and polyneuropathy
- Neuroimaging, CSF analysis, assessment of presence of APL antibodies are recommended. Management of NPSLE depends on the underling pathogenesis whether it is an ischemic or an inflammatory pathway. (LOE 3, GOR C)
- Glucocorticoids and immunosuppressive therapy are recommended for inflammatory neuropsychiatric manifestations (e.g., psychosis, aseptic meningitis, myelitis, cranial, and peripheral neuropathies) after exclusion of non-SLE causes. (LOE 3, GOR C)
- In case of ischemic NPSLE with history of APS long-term anticoagulation is recommended. Warfarin is preferred to direct oral anticoagulants in patients with thromboembolic antiphospholipid syndrome with high-risk antiphospholipid antibody. Aspirin 100 mg/day is recommended in absence of APS. (LOE 3, GOR B)
- In most patients, inflammatory and ischemic NPSLE coexist a combination of therapies, including immunosuppressive, anticoagulation, and antiplatelet therapy can be used. (LOE 4, GOR C)
- The combination of methylprednisolone and intravenous cyclophosphamide is the treatment of choice and can be effective if used promptly
- Rituximab could be considered for refractory cases 375 mg/m.^2^ weekly for 2 weeks or 1 g 2 weeks apart. (LOE 3, GOR C)
- Plasma exchange could be considered in patients with refractory neurological manifestations or IVIG 2 g/kg over 2–5 days or hematopoietic stem cell transplantation. (LOE 4, GOR C)

### Cardiac manifestation

To detect cardiac abnormalities such as pericarditis, myocardial dysfunction, and valvular lesions, echocardiography is used as a sensitive and specific investigation.

### Pericarditis

- In cases of mild, acute or chronic pericarditis, with or without effusion NSAID are recommended (LOE 3, GOR C)
- In the case of acute or chronic pericarditis with pericardial effusion, prednisone (0.5 mg/kg/day) is recommended for patients whose initial manifestation is mild to moderate pericarditis. In the case of severe or constrictive pericarditis, methylprednisolone pulses (1 g/day for 3 days) are recommended.
- In recurrent pericarditis, addition of colchicine (1 mg/day for at least 1 month) is recommended to avoid relapse. (LOE 3, GOR C)
- Surgery for pericarditis is recommended in resistant cases or cardiac tamponade not responding to medical treatment. (LOE 3, GOR C)

### Myocarditis

- For cases of myocarditis with arrhythmia, ventricular ejection fraction < 55, pulses of GC are recommended (methylprednisolone 1 g/day for 3 days) followed by prednisone (from 0.5 to 1 mg/kg/day). HCQ can be used for maintenance.
- In case of severe manifestation with arrhythmia or ventricular EF < 40%, IV Cyclophosphamide in addition to steroids is recommended for at least 3 months. If there is no response, discontinuation of the drug is recommended to prevent the risk of toxicity. If there is a response, a minimum of 6 months’ treatment is recommended. Maintenance therapy by MMF (2 g/day in divided doses) can be used or with Azathioprine (from 2 to 3 mg/kg/day) in patients who are intolerant to MMF.
- In case of complicated myocarditis when induction therapy with oral or intravenous steroids and CYC has failed, gammaglobulin at doses of 400 mg/kg/day for 5 days is recommended. (LOE 3, GOR C)

### Endocarditis

- Valvular thickening and regurge can be seen in SLE patients. Non-bacterial verrucous endocarditis can occur especially in patients with SLE and secondary APS. (LOE 3, GOR C)
- Assessment for any associated cardiac condition and disease activity is recommended.
- If discovered in the early active stage corticosteroids, prednisone 1 mg/kg/day is recommended. If the lesions become hemodynamically significant, valve surgery may be needed (LOE 3, GOR C)

### Hematological disease

#### Thrombocytopenia

Thrombocytopenia may be related to active disease, APS or a complication of immunosuppressant as azathioprine. Examination of the peripheral blood smear is recommended when microangiopathic hemolytic anemia (MAHA) or thrombotic microangiopathy (TMA) are suspected. (LOE 3, GOR B)

Treatment is considered in cases of bleeding, severe bruising, or platelet counts < 10–20 × 109/L with moderate/high doses of GC. Initial therapy with pulses of IV MP (1–3 days) is recommended. (LOE 3, GOR B) Immunosuppressives as AZA (if thrombocytopenia is not caused by AZA), MMF or cyclosporine should be introduced as GC sparing. (LOE 3, GOR B)

In severe SLE thrombocytopenia, inadequate response to high-dose GC or to avoid GC-related infectious complications, cyclophosphamide or IVIG is recommended. (LOE 3, GOR B).

In refractory cases or failure to reach a platelet count

 > 50,000/ mm^3^ or relapses, plasmapheresis or rituximab, are considered. Splenectomy can be considered. (LOE 3, GOR B)

#### Autoimmune hemolytic anemia (AIHA)

GC (1 mg/kg per day prednisone) is recommended and tapered when hematocrite rises, and reticulocytes decrease. If there is no response, consider pulse steroids, azathioprine, cyclophosphamide, or splenectomy. IVIG, danazol, MMF, and rituximab are other options for refractory AIHA (LOE 2, GOR C)

Autoimmune leucopoenia is common in SLE but barely needs treatment; careful work-up is recommended to exclude other causes of leucopoenia (especially drug-induced). (LOE 3, GOR C). Mild neutropenia (> 1.0 and < 2.0 × 109 needs no treatment with observation of CBC. If neutropenia < 1.0 × 109, primary hematological disease, infection or drug toxicity should be excluded. If immune mediated should (neutropenia < 1000 with fever or infection), granulocyte-colony stimulating factor can be given starting with a dose of 300 μg/day and continuing with the minimum effective dose to achieve a neutrophil count above 1000/l. Prednisolone 1 m/kg/day can be considered. In refractory cases, rituximab and stem cell transplantation can be considered. (LOE 3, GOR C)

Q. What are the recommendations for management of acute emergencies in patients with SLE? [35–39] (Fig. 4)

Mean rate ± SD: 8.27 ± 1.8, % of agreement: 90.9%, LOE: H

#### Acute neurologic involvement

- In acute neuropsychiatric manifestation including psychosis, aseptic meningitis, cerebral vasculitis, and neuropathies, the use of high dose GCs plus CYC is recommended over GCs alone as first line treatment. GCs plus RTX is recommended in refractory cases.
- In cases with cerebral vasculitis or inflammatory NPSLE with complete clinical response maintenance therapy for 1 year using azathioprine 2 mg/kg/day or MMF 3 g/day or cyclosporin 500 mg/day and oral prednisolone. (LOE 2, GOR C)
- In cases with cerebral vasculitis or inflammatory NPSLE with incomplete clinical response extended duration of GC and CYC for 18 months. (LOE 2, GOR C)

#### Systemic vasculitis

Treatment of systemic vasculitis is usually treatment tailored according to system affected but in life-threating condition or severe lupus vasculitis, intravenous high-dose corticosteroids, cyclophosphamide, rituximab, intravenous immunoglobulin, and/or plasmapheresis are considered. (LOE 3, GOR B)

### Acute profound thrombocytopenia with a thrombotic thrombocytopenia purpura (TTP)–like syndrome

- Pulses of intravenous methylprednisolone is recommended. In case of life-threatening bleeding or infection IVIG with or without GCs is recommended.
- Rituximab and/or IVIG should be considered in refractory cases. Tissue plasma exchange can be used. Methylprednisolone is recommended until cessation of the hemorrhage.
- Plasmapheresis is a treatment for cases where patients responded inadequately to high doses of corticosteroid and cyclophosphamide therapy. (LOE 2, GOR C)
- Eltrombopag is a thrombopoeitin (TPO) receptor agonist that activates TPO surface receptor on the megakaryocytes increasing platelets production and is approved for treatment of ITP. (LOE 2, GOR C)
- Eculizumab can be used in refractory cases.
- Splenectomy can be done in refractory cases. (LOE 2, GOR C)

#### Rapidly progressive glomerulonephritis

- Induction management with CYC 750 mg to 1 g/m.^2^ of body surface area is recommended/month, or, MMF (from 2 to 3 g/day for 6 months) in addition to pulses of methyl prednisone 1 g/day for 3 days with gradual tapering according to outcome). (LOE 2, GOR B)
- Tacrolimus is recommended at doses of 0.1–0.15 mg/kg/day orally in two divided doses, should be considered as an alternative induction treatment to iv CYC or MMF. (LOE 2, GOR B)
- Rituximab for refractory cases can be used.
- Maintenance treatment with MMF (from 2 to 3 g/day), azathioprine (from 2 to 3 mg/kg/day) and prednisone at a tapering dose is recommended. (LOE 2, GOR B)
- Permanent HCQ is recommended to reduce the possibility of renal relapse.
- ACE-I is recommended as antiproteinuric agents. Control of blood pressure, lipid profile, and weight loss in obese patients are essential measures. (LOE 2, GOR B)

#### Pulmonary hemorrhage

IV moderate-to-high dose GC in combinations with immunosuppressive therapy such as Cyclophosphamide or Rituximab with or without plasmapheresis is recommended. IVIG is recommended in refractory cases. (LOE 3, GOR C)

Q. What are the recommendations for management of infection? [40–45]

Mean rate ± SD: 8.81 ± 0.39, % of agreement: 100%, LOE: H

### Prevention of infectious agents

Baseline screening for hepatitis markers, human immunodeficiency virus, and latent tuberculosis should be part of routine clinical practice in endemic and developing countries and is of particular importance before the commencement of immunosuppressives and/or biologic agents. (LOE 3, GOR B)

Prophylaxis against pneumocystis jiroveci is warranted in patients at high risk of infection, including those on high doses of immunosuppressive especially when combined with high GC doses. (LOE 3, GOR C)

Vaccinations should be implemented according to the international guidelines for vaccination of patients with rheumatic diseases. (LOE 3, GOR B)

### Prevention of infection in special clinical situations

- Patients with valvular vegetations should receive prophylaxis for endocarditis according to the guidelines. (LOE 4, GOR C)
- Patients who underwent splenectomy should receive vaccination for pneumococci and meningococci
- Prophylaxis against COVID infection includes following the advised national control precautions such as social distancing. COVID vaccination should be implemented to all SLE patients in accordance with the international guidelines of COVID vaccination. (LOE 3, GOR C)

Treatment of SLE during active infection (LOE 3, GOR B)

Glucocorticoids tapering of GC is not recommended to avoid adrenal suppression.

Escalation of oral GC dosage is not recommended.

However, in case of life or organ threatening flare, low-dose MP might be as efficacious but safer than high dose MP. (LOE 3, GOR C)

Immunosuppressive and immunomodulatory agents immunosuppressive(s) or biologic agents should be withheld.

Whereas IVIG could be administered as a rescue immunomodulatory therapy in organ- or life- threatening flares with concomitant infections. (LOE 3, GOR B)

Q. What are the recommendations for treating comorbidities [46–50]?

Mean rate ± SD: 8.5 ± 1.71, % of agreement: 95.5%, LOE: H

- SLE patients are at higher risk of developing various comorbidities owing to several modifiable and non-modifiable factors such as underlying immunologic aberrations, the burden of persistent or recurrent disease activity, and GC and IS administration. Comorbidities usually accompanying SLE include infections and several vascular, endocrinal, and metabolic diseases (LOE 3, GOR B)
- Achieving treatment targets (LDA or remission) is key to minimize systemic inflammatory burden, attain lowest GC and IS doses, and decrease accrual damage. (LOE 3, GOR B)
- Unless contraindicated, HCQ should be administered to all SLE patients, irrespective of the disease activity state and nature of comorbidity. (LOE 2, GOR B)
- Regular screening for comorbidities among all SLE patients is mandatory guided with their clinical context/profile and risk factors.
- Management of SLE and possible associated comorbidities should commence with patient’s education about the disease, importance of medications’ adherence, life-style modifications such adequate diet intake, regular exercise, and avoiding alcohol and smoking. (LOE 3, GOR C)
- A multidisciplinary approach is warranted for risk factors’ assessment and mutual comorbidities’ treatment. (LOE 3, GOR B)

Cardio- and cerebra-vascular comorbidities (LOE 2, GOR B).

- Stringent diabetes mellitus and hypertension control has positive cardio- and cerebro-vascular outcomes. (LOE 3, GOR C)

Prophylactic administration of lipid lowering agents is not recommended and SLE patients with dyslipidemia should be managed as the general population (LOE 3, GOR C)

Primary thromboprophylaxis with ASA among SLE patients with negative antiphospholipid serology should be in accordance with the guidelines for the general population. (LOE 3, GOR B)

### Bone and muscle comorbid conditions

A ***Osteoporosis***
  Administration of daily calcium (1000–15,000 mg/day) and vitamin D (800–2000 IU/day) is mandatory for all patients receiving glucocorticoids.
  Patients receiving prednisolone ≥ 7.5 mg (or equivalent) for ≥ 3 months should receive antiresorptive agents, with the choice of the adequate antiresorptive being tailored according to each patient. (LOE 2, GOR B)
B ***Osteonecrosis***
  Conservative measures for symptomatic ON include pain management taking into consideration comorbidities.
  Cox-administration of cyclo-oxygenase-2 selective inhibitors rather than other NSAIDs is preferred especially with concomitant GC intake. Administration of concomitant proton pump inhibitors is advised.
  Patients needing surgical intervention should be cared for perioperatively in accordance with the guidelines including medications’ adjustment. (LOE 2, GOR C)
C ***Myopathy***
  Exercises could be beneficial to obviate and treat GC-induced myopathy.
  Aerobic exercises.
  Monitored resistance training which focuses on low back and whole-body resistance exercises. (LOE 2, GOR C)

#### Ophthalmological affection

Keratoconjunctivitis sicca, episcleritis, scleritis, and most importantly retinal vasculitis can be seen in patients with SLE. Side effects of drugs commonly used in SLE (corticosteroids, and HCQ) include ophthalmologic infection(s), glaucoma, and/or subcapsular cataract, and central serous maculopathy can be seen. (LOE 2, GOR C)

* Prognosis:

Q. What are the poor prognostic markers? What are the recommendations for patients with poor prognostic markers? [51–55]

Mean rate ± SD: 8.77 ± 0.52, % of agreement: 100%, LOE: H

Poor prognostic factors include

- Sociodemographic poor prognostic factors include male gender, smoking or alcoholism, juvenile disease-onset, low educational status, lack or insufficient health insurance, poor income, and poor medication adherence. (LOE 2, GOR C)
- Clinical poor prognostic markers include number of ACR or EULAR criteria, high baseline disease activity or damage, persistent high disease activity throughout the course of the disease, recurrent flares, major organ involvement, presence of antiphospholipid syndrome, and coexisting comorbidities including infections. (LOE 2, GOR C)
- Laboratory poor prognostic markers include presence of antiphospholipid antibodies, and anti-DNA. (LOE 3, GOR C)
- Treatment poor prognostic factors include: glucocorticoid dosage and duration, and immunosuppressives use (LOE 3, GOR C)
- Specific organs poor prognostic factors include

Renal: hypertension at presentation, elevated baseline serum creatinine and hematuria, lack of achieving EULAR/ERA-EDTA response at one year, poor histopathologic features which include the presence of crescents and histopathologic features of chronicity such as glomerular sclerosis, tubule-interstitial fibrosis or tubular atrophy, and vascular lesions (LOE 2, GOR C)

Neurologic: number of NP events (≥ 2), diffuse NP events*, anti-phospholipid antibodies (LOE 3, GOR C)

Cardiopulmonary: pulmonary hypertension, and shrinking lung syndrome (LOE 3, GOR C)

Recommendations for patients with poor prognostic markers

Patients with poor prognostic factors are more likely to have direct increased accrual damage, and a higher probability to inadequate treatment response; hence, SLE patients with poor prognostic factor(s) need closer follow-up and potential initial aggressive or more rapid escalation of immunosuppressive therapy might be necessary. (LOE 3, GOR B)

## Discussion

SLE continues to be a challenging and disabling disease, due to its chronic nature, associated multisystem affection and variable serological and laboratory test results. SLE has also a major negative impact on the individual patient’s health and lifestyle. In addition, the disease is prone to relapses and flares of disease activity resulting in substantial morbidity due to accumulated organ damage. Unfortunately, in spite of some advances in treatment approaches with consequent better survival data over the past few decades [56], patients living with SLE remain at high risk of dying, on average 25 years, earlier than the mean for their peers whether men or women [57]. Prompt diagnosis and appropriate timely management as well as regular monitoring are vital to minimize such risk of morbidity or mortality [58]. The development of new classification criteria, as well as better understanding of the disease pathogenesis associated with aberrant regulation of both innate and adaptive immune responses [59], causing excessive production of auto-antibodies, have paved the way for early diagnosis of the disease, and development of novel therapies that are more effective and less toxic. Therefore, it is essential to develop an up-to-date comprehensive recommendation capitalizing on the strengths of and experience from the previous projects and to optimize the treatment aspects to be consistent with the current evidence. The aim is to improve the outcomes of this potentially life-threatening disease.

The objective of this guideline was to develop a list of management recommendations for adult patients living with SLE in Egypt. So far, there has not been any Egypt-based guidelines developed for the management of Egyptian patients living with SLE. The developed list was intended to cover the disease management and monitoring, considering the different disease activity status, whether mild, moderate, or severe. The management statements developed in this work were based on thorough review of the literature and consensus agreement of a national expert panel. Results of this study revealed that the level of agreement ranged from 95.5 to 100%. Reflecting the strength of the agreement on the statements which cover all the important aspects of the disease. While primarily rheumatologists were the main target of this guideline, it was also developed aiming at nephrologists, dermatologists, emergency medicine physicians, immunologists, general practitioners, and trainees who might also seek guidance for their management strategy.

The developed guidelines were broadly in agreement with the recently published guidelines namely the EULAR update on the diagnosis and management of systemic lupus erythematosus [9] as well as The British Society for Rheumatology guideline for the management of systemic lupus erythematosus in adults [60]. The guideline covers management strategy, treatment targets as well as adjunct therapy. The severity in SLE was determined based on (a) the presence of major organs affection or organ-threatening risk; (b) the occurrence of concomitant activity attributed to multiple non-major organs; and (c) the requirement for treating with high glucocorticoids doses and/or immunosuppressive therapy. Medical therapy was suggested as first line options with alternative protocols for non-responsive or refractory cases. On the other hand, this guideline did not include the evidence for the diagnosis of SLE. In fact, it was developed based on the EULAR/ACR classification criteria of the disease [59]. It did not cover also the management of pregnant lupus patients, as well as patients living with lupus nephritis as these have been covered in separate guidelines [60]. It also did not cover management of children living with SLE. However, as the disease tends to affect adolescents after puberty, most of the recommendations are likely to be suitable for this cohort of patients with appropriate dose modifications. The management of associated comorbidities such as cardiovascular risk, infection, osteoporosis whether induced by the disease or its medical therapy, e.g., steroids, infections, and risk of cancer have not been discussed in full details in this guideline. This was based on the fact that these disorders have their own national and international recommendations for management. However, self-management has been endorsed in this guideline for the treatment of disease-associated complications such as fatigue, headache, and sleep disturbance. Management of thrombosis has been included in this guideline only for the patients who met the anti-phospholipid syndrome criteria [61].

The guideline recommended the treat-to-target management approach tailored to the patient’s specific medical status, disease activity and associated comorbidities. It also endorsed ‘multitargeted’ therapy as well as organ-specific outcome measures to be able to ensure achievement of the treatment target(s). To be able to implement the treat-to-target approach and plan for the appropriate treatment, it has been recommended to score the individual patient’s disease activity, which was determined using an SLE disease activity score, level of functional disability as well as the current steroids dose. Consequently, the patients are stratified to mild, moderate, or severe [59]. Patient reported outcomes has been endorsed as an approach to monitor the patients’ response to therapy. Worsening disease activity has been identified as flare , which consequently can be categorized as mild, moderate or severe [62]. This comes in agreement with the EULAR treat-to-target recommendations for SLE [63]. The approach of “treat-to-target” has been efficiently implemented in several chronic diseases whether rheumatic or non-rheumatic. It has also been implemented in other inflammatory arthritic conditions in Egypt [64, 65]. Identifying appropriate therapeutic targets and chasing these systematically has led to improved-quality care for patients with these diseases and valuable guidance for healthcare professionals as well as administrators [63].

As the terminology suggests, evidence-based medicine relies on identifying the evidence and using that evidence to make clinical decisions [66]. Furthermore, the grading system provides a significant component in evidence-based medicine and helps in the clinical decision-making process. In this work, Oxford Centre for Evidence-based Medicine (CEBM) system was implemented. This is in agreement with the EULAR guidelines for several inflammatory rheumatic disorders [67]. In contrast, the ACR implement the Grading of Recommendations Assessment, Development and Evaluation (GRADE) [68]. In contrast to the GRADE which uses four levels for quality of evidence: high, moderate, low, and very low; the Oxford levels of evidence include 10 categories (Table 2). These levels imply a gradient of confidence in estimates of treatment effect and thus a gradient in the consequent strength of inference [69]. While GRADE provides a systematic and transparent approach to assessing the certainty of evidence and strength of recommendations, it is important to acknowledge that using GRADE will commonly involve some subjective judgments, and assessments may vary between individuals [70, 71]. This is supported by the finding that inter-rater agreement for GRADE assessments by different, untrained individuals is limited [72, 73].

Guidelines help clinicians translate best evidence into best practice [74]. However, it does not imply a legal obligation. It is important to highlight that adherence to management recommendations will not guarantee an effective result for each patient in every clinical scenario. The decisive assessment should be carried out by a rheumatologist responsible for the clinical decision-making and considering the individual patient’s medical status, favorite options, values and priorities. Recommendations stated in this guideline have been set up based on the best clinical evidence. Clinical practice guidelines aim to provide a frame on how to enhance the suitability and quality of care, to improve the interventions’, cost-effectiveness, to categorize relevant research pathways and to act as a tool for education. Based on the level of evidence and strength of the recommendations, the guideline is intended to help inform clinical decision-making [75].

Limitations of the guideline: the limited comparative evidence to advise the appropriate therapeutic choice is a limitation to the current published guidelines. Consequently, therefore, indirect comparisons among therapies/trials were used for the purpose of this work. Though this guideline signifies the best data available at the time of preparing this report, attention should be exerted in interpreting the data; future studies may authorize modification of the conclusions or recommendations included in this study.

In conclusion, the diagnosis and treatment of SLE patients in Egypt has often been inconsistent, with those seeking a diagnosis frequently facing delays and experiencing uncertainty about their treatment plan. We hope that the implementation of the guideline will lessen the current challenges in both the management as well as monitoring, and result in earlier access to appropriate therapies, reducing flares and, ultimately, giving a better quality of life for the patients. It endorses patient reported outcome measures as a monitoring approach with multi-disciplinary team backup.

## Data Availability

The data will be available upon reasonable request.

## Abbreviations

ACE-I: Angiotensin-converting enzyme inhibitors
ACR: American College of Rheumatology
AIHA: Autoimmune haemolytic anemia
Anti-DNA: Anti–double-stranded deoxyribonucleic acid
APL: Anti-phospholipid antibodies
ASA: Aspirin
BMD: Bone mineral density
CLE: Cutaneous lupus erythematosus
COVD: Coronavirus disease
CS: Corticosteroids
CYC: Cyclophosphamide
eGFR: Estimated glomerular filtration rate
EULAR: European Alliance of Associations for Rheumatology
EULAR/ERA-EDTA: European League Against Rheumatism/European Renal Association-European Dialysis and Transplant Association (EULAR/ERA-EDTA),
FRAX: Fracture risk assessment tool
HRQoL: Health-related quality of life
IA: Intra-articular
ICU: Intensive care unit
IM: Intramuscular
ITP: Idiopathic thrombocytopenic purpura
IV: Intra-venous
IVIG: Intravenous immunoglobulins
LDAS: Low disease activity state
Lupus QoL: Lupus quality of life
mAB: Monocolonal antibody
MAHA: Microangiopathic hemolytic anemia
MMF: Mycophenolate mofetial
MP: Methylprednisolone
MTX: Methotrexate
NP: Neuropsychiatric
NPSLE: Neuropsychiatric systemic lupus erythematosus
NSAID: Non-steroidal-anti-inflammatory
ON: Osteonecrosis
PROM: Patients reported outcomes measures
RTX: Rituximab
SCLE: Subacute cutaneous lupus erythematosus
SLE: Systemic lupus erythematosus
SLEDAI: Systemic Lupus Erythematosus Disease Activity Index
SLEDAI-2K: Systemic Lupus Erythematosus Disease Activity Index 2000
SLICC/ACR DI: The systemic lupus international collaborating clinics American College of Rheumatology Damage index
T2T: Treat-to-target
TMA: Thrombotic microangiopathy
TTP: Thrombotic thrombocytopenia purpura
